# Supplementary material for: SALL3 expression balance underlies lineage biases in human induced pluripotent stem cell differentiation
Source: Nat Commun. 2019 May 15;10:2175. doi: 10.1038/s41467-019-09511-4 (PMC6520385; doi:10.1038/s41467-019-09511-4)
Supplement: Supplementary file 1 — Supplementary Information [file 41467_2019_9511_MOESM1_ESM.pdf]

Supplementary information

SALL3 expression balance underlies lineage biases in human induced pluripotent stem cell differentiation

Takuya Kuroda et al.

Supplementary Figure 1. Heat map of differentiation marker expression.

A

Detector	201B7	253G1	409B2	DYR0100	HYR0103	mc-iPS	R-1A	R-2A	R-12A	Tic
ABCG2	-0.26	-0.73	-0.14	-0.02	0.13	-0.90	-0.64	0.41	2.60	-0.44
APOE	0.03	-0.78	1.18	0.20	0.43	-1.54	-0.69	1.82	0.12	-0.77
CD44	-0.36	-0.68	0.27	0.42	0.34	-0.98	-0.54	2.45	-0.02	-0.90
CDH2	0.94	1.26	-0.27	-0.85	-0.87	1.60	0.61	-0.61	-0.81	-1.01
CRABP2	1.97	1.44	-0.19	-0.48	-0.87	0.32	0.17	-0.75	-0.66	-0.94
EN1	-0.12	-0.34	-0.28	-1.13	-0.76	0.09	2.38	-0.71	0.86	0.01
FAS	0.68	1.22	0.44	-0.99	-0.79	-0.60	0.70	1.38	-0.56	-1.48
FGF5	1.52	-0.06	-0.43	-0.79	-0.87	0.43	0.01	-0.69	1.87	-0.99
FGFR2	0.23	-0.43	1.62	-0.29	0.15	-1.24	-0.24	1.54	0.08	-1.43
FOXD3	1.35	1.26	-0.65	-0.31	-0.69	1.51	-0.10	-0.30	-1.16	-0.91
FUT4	0.45	-0.55	0.57	-0.54	0.10	-0.70	0.08	-0.40	2.33	-1.34
GATA2	-0.46	-0.54	-0.31	-0.29	0.03	-0.66	-0.59	0.21	2.73	-0.14
GATA3	-0.31	-0.64	-0.14	0.01	0.18	-0.88	-0.56	-0.27	2.70	-0.10
GBX2	-0.12	1.57	-0.53	-0.59	-0.69	0.41	1.94	-0.36	-0.78	-0.86
HAND1	0.31	-0.53	1.86	-0.27	0.28	-1.33	-0.93	1.10	0.47	-0.96
ICAM1	0.40	-0.24	0.01	2.04	-0.62	-1.39	0.22	1.02	-0.40	-1.04
IRF6	1.99	-0.74	-0.12	0.75	-0.38	-0.78	0.15	-0.75	1.06	-1.19
ITGA4	1.38	1.72	-0.61	-0.91	-0.85	0.42	0.35	0.38	-0.85	-1.02
ITGA6	0.10	-0.21	2.08	-0.31	0.25	-1.12	-0.93	1.26	-0.28	-0.84
ITGB1	0.89	-0.20	0.67	-0.17	0.41	-1.61	-0.30	1.12	0.86	-1.68
MAP2	0.41	1.02	-0.49	-0.76	-0.78	1.27	1.70	-0.81	-0.76	-0.80
MAPT	0.74	0.18	-0.66	-0.53	-0.74	1.31	1.93	-0.74	-0.75	-0.75
MCAM	1.35	2.12	-0.30	-1.04	-0.62	-0.07	-0.14	0.14	-0.53	-0.91
MNX1	0.06	0.44	-0.70	-0.41	-0.65	0.75	2.42	-0.48	-0.58	-0.86
NCAM1	1.03	0.47	0.51	-0.80	-0.84	0.22	1.88	-0.32	-0.84	-1.31
NEFL	0.60	0.03	-0.55	-0.59	-0.68	0.85	2.34	-0.62	-0.72	-0.67
NES	1.10	0.98	0.04	-1.18	-0.67	1.10	1.21	-0.94	-0.64	-1.01
NEUROG3	-0.75	-0.11	0.24	-0.45	-0.82	0.11	2.34	0.92	-0.57	-0.91
NGFR	1.56	1.43	-0.13	-0.50	-1.02	0.26	0.82	-0.24	-1.07	-1.11
NOG	0.88	-0.16	0.02	-0.99	-0.70	0.06	0.54	0.13	1.89	-1.68
NOTCH1	1.27	0.44	0.77	-0.69	-0.83	0.33	0.97	0.53	-1.10	-1.70
OTX2	1.27	1.77	-0.04	-0.90	-0.85	1.02	-0.10	-0.94	-0.67	-0.57
PAX3	0.98	0.74	-0.61	-0.77	-0.79	1.48	1.35	-0.79	-0.79	-0.79
PAX6	0.92	1.75	-0.32	-0.77	-0.79	1.37	0.20	-0.80	-0.78	-0.78
PAX7	0.62	0.45	-0.57	-0.75	-0.77	1.53	1.71	-0.78	-0.72	-0.73
PDGFRA	0.27	-0.38	1.54	-0.10	-0.12	-1.32	0.28	1.58	-0.35	-1.40
SNAI2	0.00	-0.52	1.44	-0.40	-0.04	-1.22	-0.16	1.75	0.48	-1.31
SOX10	1.66	1.59	-0.67	-0.70	-0.70	0.84	0.15	-0.71	-0.73	-0.72
SOX2	0.81	1.32	0.04	-1.06	-0.89	1.85	-0.68	-0.27	-0.79	-0.34
SOX9	1.76	1.00	-0.32	-0.88	-0.73	1.15	0.32	-1.08	-0.33	-0.90
SYP	1.19	0.38	-0.65	-0.76	-0.75	1.77	1.05	-0.76	-0.75	-0.71
TDGF1	-0.86	-0.62	-0.03	1.33	0.26	-1.17	-1.15	1.66	-0.17	0.75
TH	-0.18	-0.28	-0.36	-0.35	-0.41	-0.04	2.83	-0.38	-0.41	-0.41
THY1	-0.05	-0.07	0.02	0.46	-0.07	-1.28	-0.60	2.50	-0.25	-0.68
TUBB3	1.44	0.51	-0.64	-0.84	-0.81	1.58	0.90	-0.67	-0.67	-0.81

High | Medium | Low

B

Detector	201B7	253G1	409B2	DYR0100	HYR0103	mc-iPS	R-1A	R-2A	R-12A	Tic
ABCG2	1.09	0.40	1.26	1.44	1.65	0.15	0.54	2.06	5.25	0.82
ACTC1	1.07	0.66	0.40	0.30	0.61	0.48	0.31	0.50	1.25	0.41
ADIPOQ	1.22	1.20	7.47	3.89	0.87	1.99	6.78	2.09	1.02	0.54
ANPEP	1.07	0.34	1.63	1.18	1.23	0.09	1.14	2.74	0.84	0.29
BMP2	1.01	0.73	1.01	0.90	0.69	0.57	0.77	0.89	0.50	0.29
CD34	1.08	0.31	2.47	0.51	1.23	0.09	1.29	2.44	0.67	0.13
CD36	1.09	0.59	1.78	0.51	0.58	0.33	1.99	2.74	1.05	0.23
CD4	1.04	1.91	0.83	0.55	0.67	0.51	2.30	1.64	0.61	0.12
CD44	1.12	0.60	2.15	2.40	2.26	0.09	0.83	5.74	1.67	0.23
CDH2	1.06	1.22	0.44	0.14	0.14	1.39	0.89	0.27	0.16	0.06
CDH5	1.19	0.56	2.53	0.93	1.98	0.10	1.24	3.34	12.08	1.18
CEACAM1	1.20	0.73	1.98	2.33	1.05	0.27	2.06	4.73	5.95	0.47
DLL1	1.03	1.05	0.50	0.27	0.33	1.62	1.13	0.17	0.39	0.38
EOMES	1.20	1.03	0.28	0.43	1.27	0.82	0.74	0.84	0.81	0.19
FGF5	1.33	0.56	0.38	0.20	0.17	0.80	0.59	0.25	1.50	0.11
FOXC1	1.03	0.72	0.65	0.20	0.24	0.33	1.18	0.13	0.25	0.18
FUT4	1.07	0.73	1.11	0.73	0.95	0.68	0.94	0.78	1.71	0.46
GATA2	1.10	0.71	1.76	1.86	3.26	0.20	0.50	4.09	15.37	2.52
GATA3	1.04	0.59	1.27	1.49	1.72	0.27	0.71	1.10	5.18	1.34
GATA4	1.02	0.62	0.67	0.92	0.74	0.20	0.71	1.10	0.57	0.17
HAND1	1.09	0.56	2.05	0.72	1.07	0.06	0.31	1.58	1.19	0.29
HHEX	1.02	3.07	1.54	0.97	1.11	0.51	1.06	1.26	1.41	1.21
ICAM1	1.18	0.88	1.00	1.94	0.71	0.35	1.10	1.47	0.81	0.51
INHBA	1.24	0.98	2.32	5.63	1.75	0.24	1.90	6.11	6.22	1.07
ITGA4	1.11	1.26	0.21	0.08	0.11	0.68	0.64	0.66	0.10	0.03
ITGA6	1.06	0.91	2.03	0.86	1.13	0.46	0.56	1.63	0.88	0.60
ITGAL	1.09	1.17	2.23	1.74	2.16	0.29	2.47	3.52	1.12	0.76
ITGAM	1.10	0.91	2.16	2.61	1.59	0.69	2.03	5.26	1.47	0.65
ITGAV	1.04	1.09	0.85	0.88	0.78	0.53	0.97	1.01	1.61	0.40
ITGAX	1.11	0.84	1.49	0.97	0.56	0.59	0.49	1.25	0.52	0.40
ITGB1	1.03	0.75	0.97	0.76	0.90	0.38	0.72	1.09	1.02	0.37
ITGB3	1.16	0.48	2.45	1.67	1.19	0.13	1.59	2.76	2.22	0.16
KDR	1.08	0.65	2.22	1.34	1.42	0.09	0.58	2.58	1.03	0.28
KIT	1.04	0.70	0.64	0.59	0.64	0.49	0.84	0.69	0.95	0.62
LEF1	1.02	0.99	0.61	0.41	0.54	1.04	1.11	0.54	0.50	0.17
MCAM	1.07	1.30	0.58	0.36	0.48	0.65	0.62	0.71	0.51	0.40
MIXL1	1.13	1.29	0.15	0.54	1.40	0.23	0.60	0.97	0.90	0.26
MME	1.34	0.88	4.47	2.71	4.24	0.18	1.36	13.55	5.01	0.41
MYOD1	1.06	1.00	1.97	0.38	0.79	0.96	0.60	1.15	0.41	0.17
MYOG	1.28	0.52	0.80	0.35	0.38	0.24	1.07	1.65	0.48	0.10
NCAM1	1.12	0.87	0.88	0.28	0.26	0.75	1.51	0.50	0.26	0.05
NES	1.02	0.98	0.68	0.29	0.45	1.02	1.06	0.37	0.46	0.34
NGFR	1.05	1.00	0.47	0.34	0.16	0.60	0.79	0.43	0.14	0.13
NODAL	1.11	1.09	0.44	0.69	1.76	0.41	0.49	2.20	0.77	1.16
NOTCH1	1.06	0.83	0.92	0.51	0.47	0.80	0.98	0.86	0.40	0.23
PDGFRA	1.07	0.72	1.76	0.87	0.86	0.21	1.08	1.79	0.74	0.17
PECAM1	1.14	0.27	3.44	0.90	1.14	0.09	1.87	4.00	0.99	0.10
RUNX1	1.15	0.54	1.51	1.11	1.29	0.40	1.21	1.76	3.37	0.82
SDC1	1.12	0.87	1.48	1.29	1.42	0.74	1.56	4.30	8.69	1.31
SPI1	1.04	1.01	4.11	2.38	3.52	0.22	3.44	8.43	1.91	0.77
SRF	1.01	0.97	0.94	0.60	0.68	0.53	0.72	0.81	0.90	0.66
STAT3	1.04	0.93	1.15	0.88	0.73	0.56	0.86	1.45	0.97	0.59
T	1.18	0.81	0.20	0.42	2.78	0.27	0.52	1.01	1.26	0.15
THY1	1.00	0.99	1.03	1.24	0.99	0.43	0.75	2.20	0.91	0.71
TNFRSF1A	1.03	0.75	1.32	0.93	0.87	0.55	0.74	1.58	1.13	0.43
TWIST1	1.19	1.12	2.14	1.29	1.11	0.47	3.58	2.25	2.01	0.19

High Medium Low

C

Detector	201B7	253G1	409B2	DYR0100	HYR0103	mc-iPS	R-1A	R-2A	R-12A	Tic
AFP	-0.39	-1.11	0.75	1.00	0.07	-1.16	0.41	1.84	-0.41	-0.99
APOE	0.03	-0.78	1.18	0.20	0.43	-1.54	-0.69	1.82	0.12	-0.77
CD44	-0.36	-0.68	0.27	0.42	0.34	-0.98	-0.54	2.45	-0.02	-0.90
CDH2	0.94	1.26	-0.27	-0.85	-0.87	1.60	0.61	-0.61	-0.81	-1.01
CDX2	0.01	-0.56	0.59	1.80	0.64	-1.65	0.82	-0.21	-0.41	-1.03
CTNNB1	1.22	0.17	1.26	-1.23	-0.37	-0.69	-0.30	0.18	1.22	-1.47
EOMES	1.21	0.74	-1.32	-0.91	1.39	0.17	-0.07	0.21	0.13	-1.56
FOXA2	0.83	-0.38	-0.23	0.67	-0.59	-0.45	2.23	-0.04	-0.86	-1.19
GATA1	-0.19	-1.00	1.44	0.48	0.34	-1.29	1.08	0.89	-0.45	-1.30
GATA4	1.13	-0.18	-0.01	0.81	0.21	-1.51	0.11	1.39	-0.33	-1.63
GATA6	0.41	-0.67	1.32	0.06	0.18	-1.45	-0.25	1.62	0.13	-1.34
GCG	-0.59	-0.74	1.51	-0.45	0.26	-0.74	1.73	0.71	-0.68	-1.01
HNF1A	-0.46	-1.15	0.49	1.29	0.20	-1.29	1.04	1.22	-0.23	-1.11
HNF1B	0.87	1.58	0.51	0.27	-0.82	-0.74	1.09	-0.40	-1.13	-1.25
ISL1	2.37	0.33	-0.56	-0.52	-0.34	0.68	-0.24	-1.24	0.19	-0.69
ITGA6	0.10	-0.21	2.08	-0.31	0.25	-1.12	-0.93	1.26	-0.28	-0.84
ITGB1	0.89	-0.20	0.67	-0.17	0.41	-1.61	-0.30	1.12	0.86	-1.68
MIXL1	0.85	1.19	-1.31	-0.45	1.43	-1.14	-0.32	0.48	0.35	-1.07
NEUROG3	-0.75	-0.11	0.24	-0.45	-0.82	0.11	2.34	0.92	-0.57	-0.91
PAX6	0.92	1.75	-0.32	-0.77	-0.79	1.37	0.20	-0.80	-0.78	-0.78
PDX1	-0.31	-0.78	-0.03	1.79	-0.62	-0.64	1.90	-0.14	-0.54	-0.63
SLC2A2	0.32	-0.87	-0.58	-0.15	-0.23	-0.78	2.31	1.06	-0.30	-0.77
SOX17	0.91	-0.51	0.20	-0.61	-0.26	-0.47	1.93	0.90	-0.53	-1.54
SOX7	-0.51	-0.94	0.22	-0.06	0.38	-1.22	-0.36	0.79	2.26	-0.56
SST	1.10	-0.09	0.11	-0.73	-0.48	-0.02	-0.52	2.26	-0.40	-1.22
SYP	1.19	0.38	-0.65	-0.76	-0.75	1.77	1.05	-0.76	-0.75	-0.71
THY1	-0.05	-0.07	0.02	0.46	-0.07	-1.28	-0.60	2.50	-0.25	-0.68

■ High □ Medium ■ Low

Supplemental Figure 1. Heat map of differentiation marker expression.

TaqMan array analysis of three germ layer differentiation marker genes in EBs (n = 6, biological replicates) derived from ten hiPSC lines. (A) ectoderm, (B) mesoderm, (C) endoderm. Results were normalized to the average of the 201B7 samples to obtain fold changes, then data were formulated using z-scoring method (standardization).

Supplementary Figure 2. EB differentiation of 606A1 and 648A1.

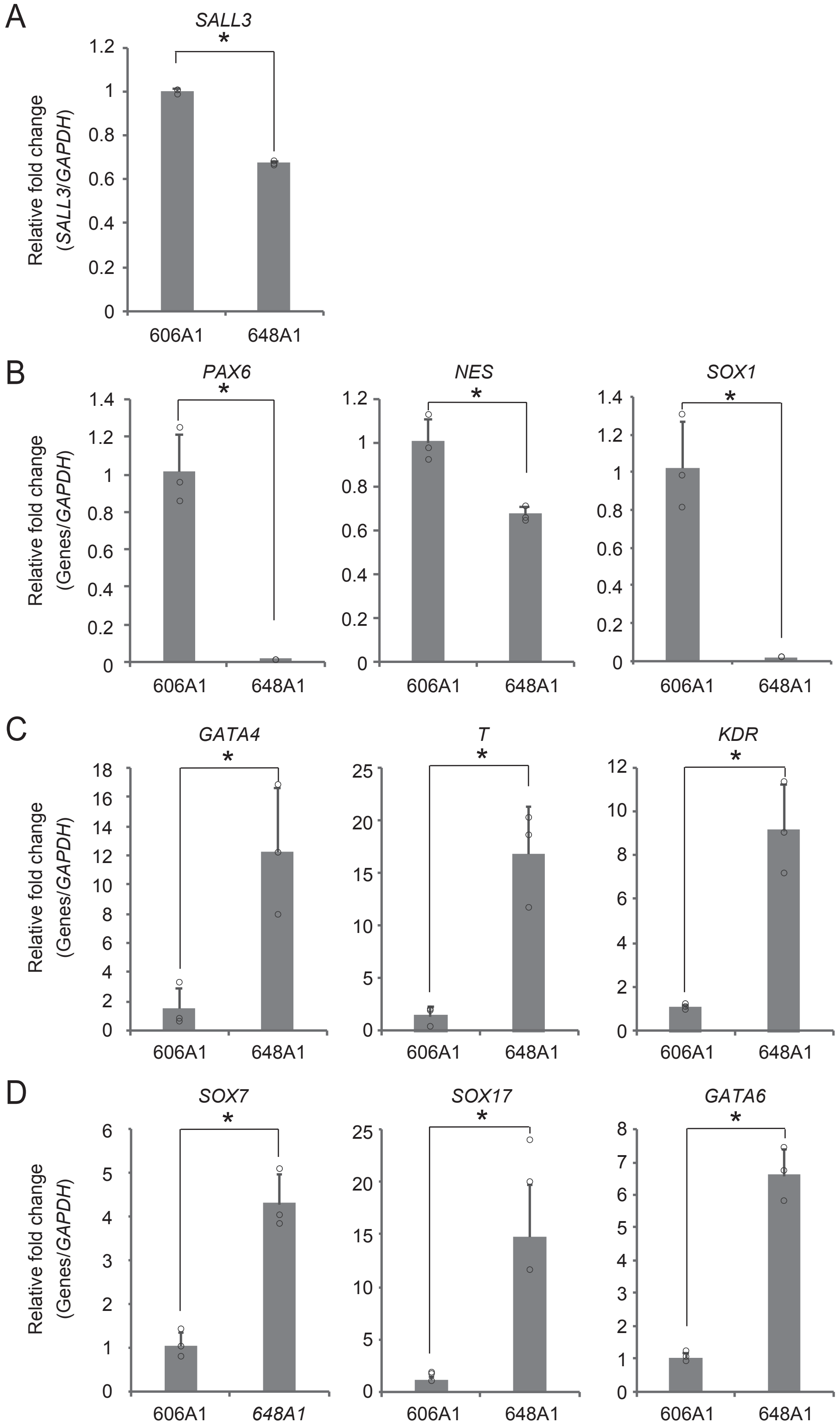

Supplementary Figure 2. EB differentiation of 606A1 and 648A1.

(A) *SALL3* expression was confirmed by qRT-PCR analysis (n=3, biological replicates). (B-D) qRT-PCR analysis of three germ layer-specific genes in EBs derived from 606A1 and 648A1 cells. (B) Ectoderm marker genes, (C) mesoderm marker genes, (D) endoderm marker genes (n=3, biological replicates). **P* < 0.01, two-sided *t* test. Error bars represent mean ± SD

Supplementary Figure 3. EB differentiation of 253G1 *SALL3* KD clone-2.

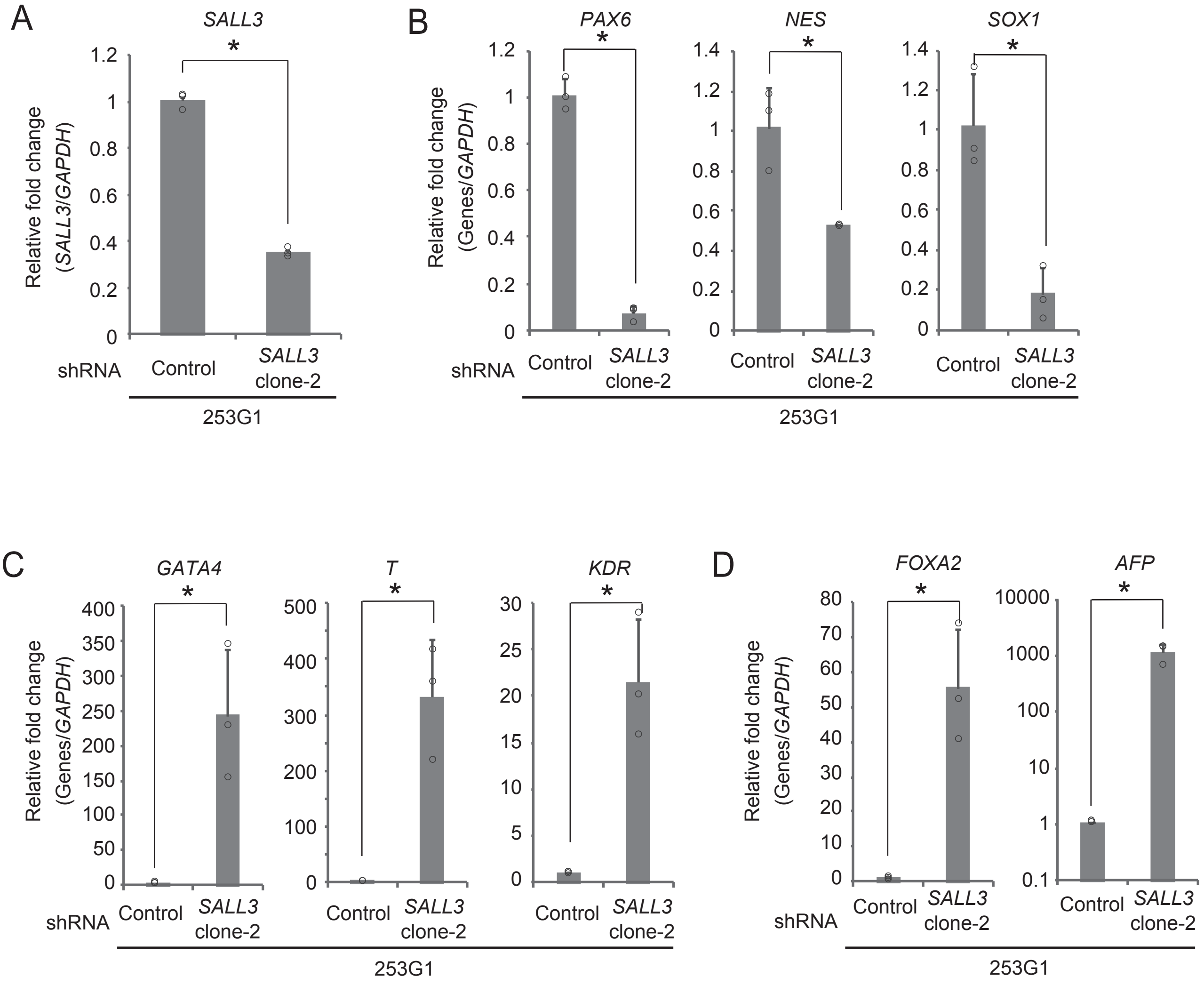

Supplementary Figure 3. EB differentiation of 253G1 *SALL3* KD clone-2.

(A) *SALL3* knockdown was confirmed by qRT-PCR analysis (n=3, biological replicates). (B-D) qRT-PCR analysis of three germ layer-specific genes in EBs derived from 253G1 SALL3 shRNA clone-2 cells and 253G1 control shRNA cells. (B) Ectoderm marker genes, (C) mesoderm marker genes, (D) endoderm marker genes (n=3, biological replicates). *$P < 0.01$, two-sided t test. Error bars represent mean ± SD

Supplementary Figure 4. Cardiomyocyte differentiation of 201B7 *SALL3* KD.

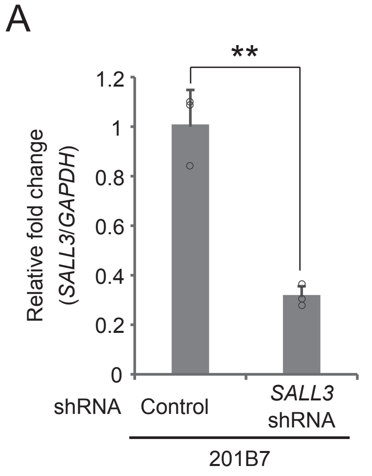

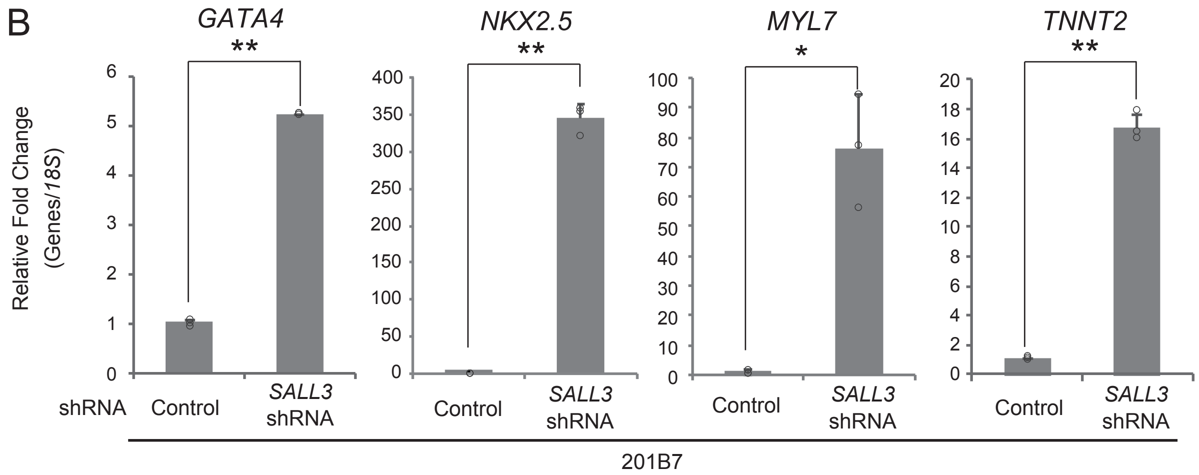

Supplementary Figure 4. Cardiomyocyte differentiation of 201B7 *SALL3* KD.

(A) *SALL3* knockdown was confirmed by qRT-PCR analysis (n=3, biological replicates). (B) qRT-PCR analysis of cardiomyocyte markers *GATA4*, *NKX2.5*, *MYL7*, and *TNNT2*. Total RNA was isolated from SALL3 shRNA cell-derived and control shRNA cell-derived cardiomyocytes (n=3, biological replicates). **P* < 0.05, ***P* < 0.01, two-sided *t* test. Error bars represent mean ± SD

Supplementary Figure 5. Neural differentiation of 201B7 *SALL3* KD.

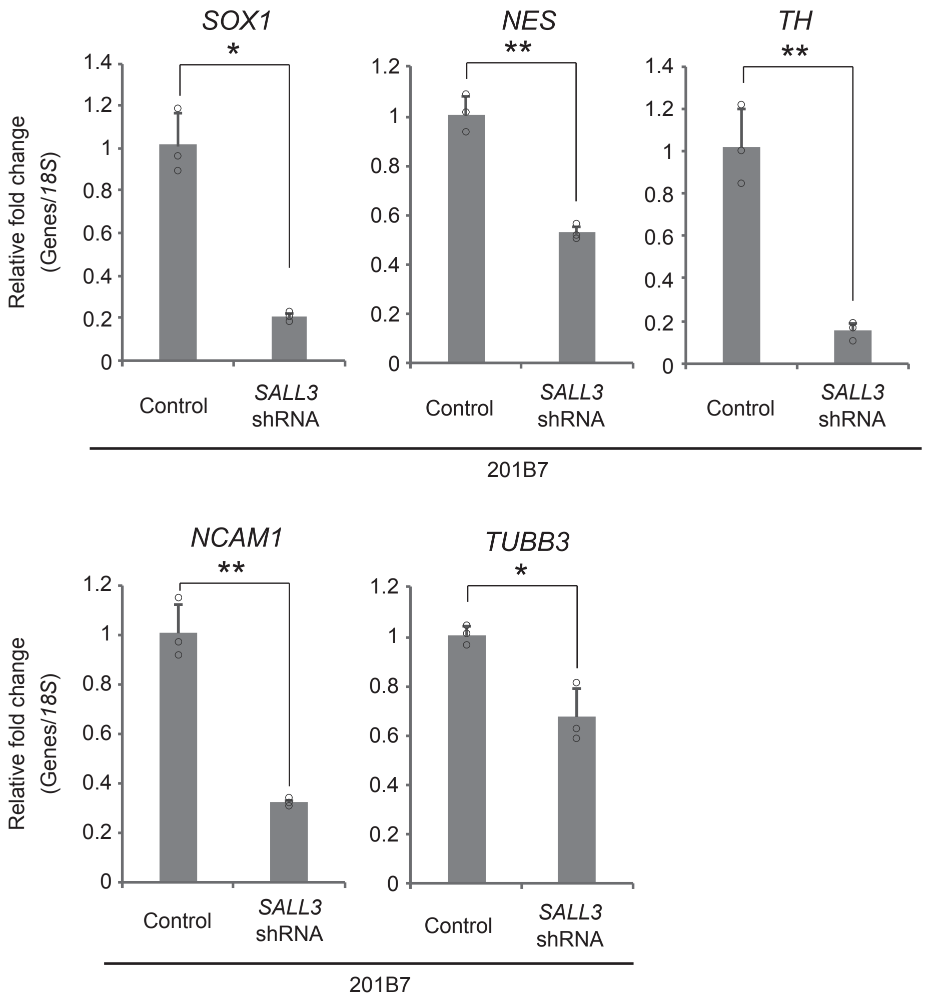

Supplementary Figure 5. Neural differentiation of 201B7 *SALL3* KD.

qRT-PCR analysis of neural cell markers *SOX1*, *NES*, *TH*, *NCAM1* and *TUBB3*. Total RNA was isolated from *SALL3* shRNA cell-derived and control shRNA cell-derived neural cells (n=3, biological replicates). *$P < 0.05$, **$P < 0.01$, two-sided *t* test. Error bars represent mean ± SD

Supplementary Figure 6. Neural differentiation of R-2A *SALL3* over expression cells.

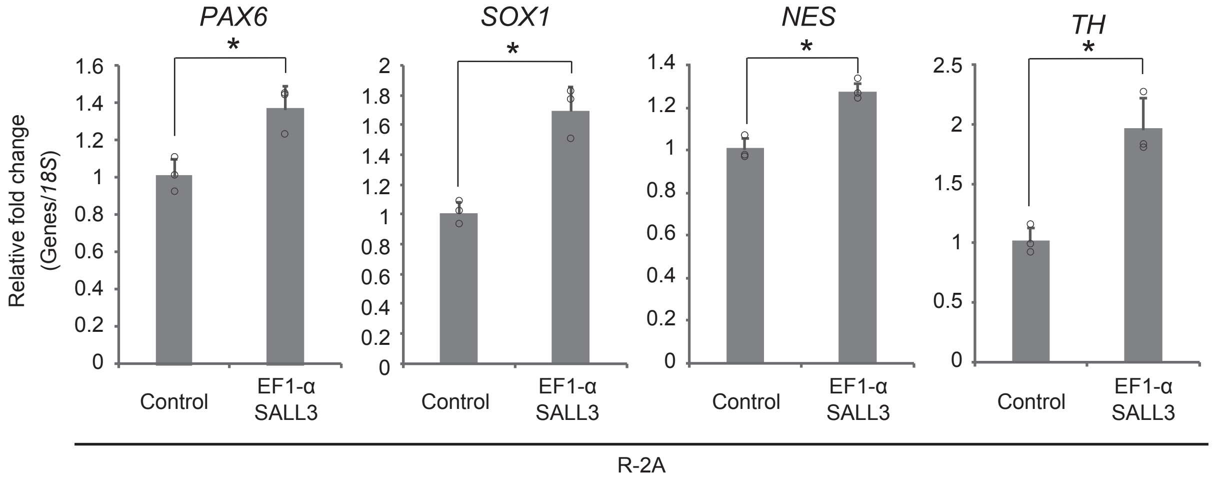

Supplementary Figure 6. Neural differentiation of R-2A *SALL3* over expression cells.

qRT-PCR analysis of neural cell markers *PAX6*, *SOX1*, *NES* and *TH*. Total RNA was isolated from R-2A EF1-α *SALL3* cell-derived and R-2A control cell-derived neural cells (n=3, biological replicates). *$P < 0.05$, two-sided *t* test. Error bars represent mean ± SD

Supplementary Figure 7. Targeted mutation of *DNMT3B* in the hiPSC line 253G1 *SALL3*$^{-/-}$.

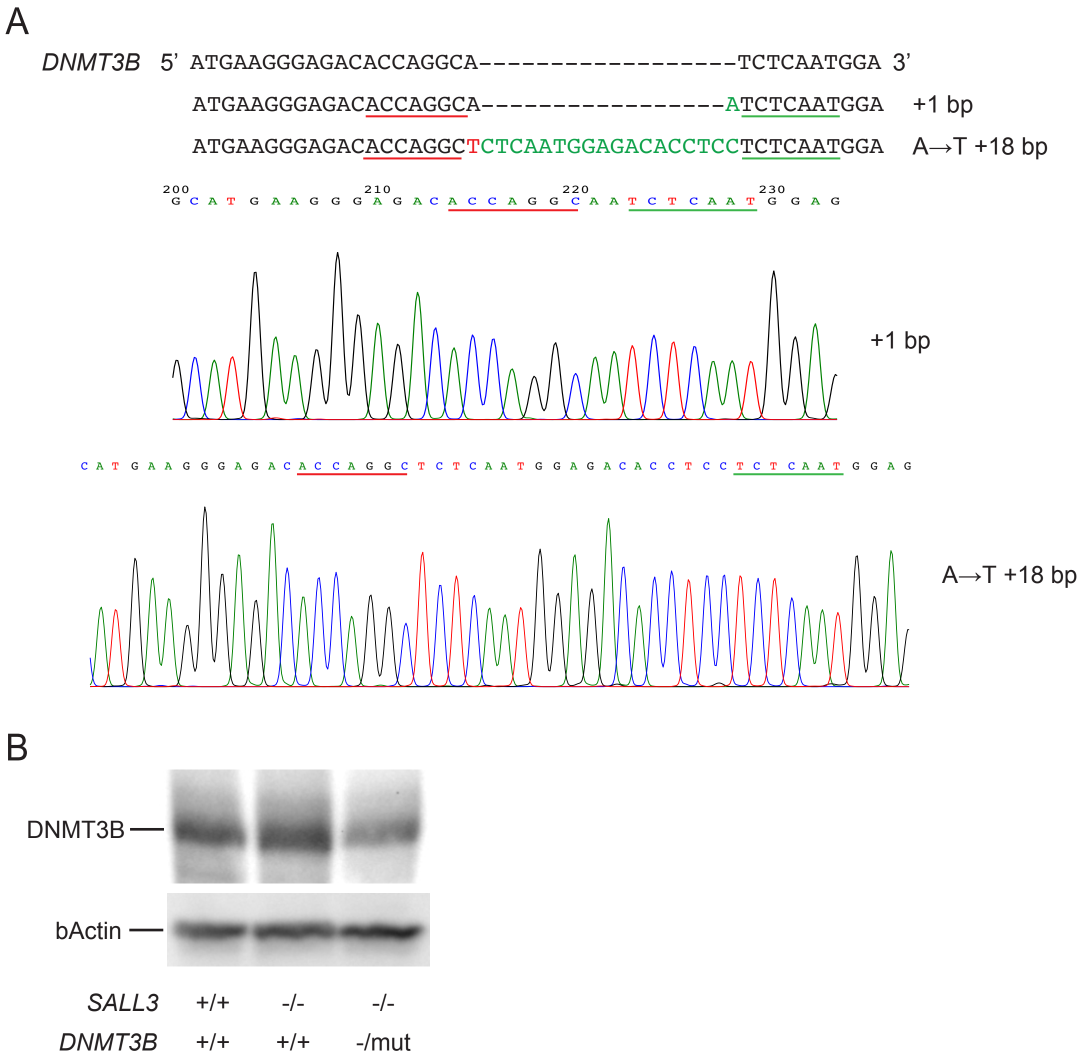

Supplementary Figure 7. Targeted mutation of *DNMT3B* in the hiPSC line 253G1 *SALL3*$^{-/-}$.

(A) Sanger sequencing information for the targeted region of representative clone of 253G1 *SALL3*$^{-/-}$ *DNMT3B1*$^{-/mut}$ cell. The colored lines under the sequence correspond to the lines under sequencing peaks. (B) Western blot analysis of the total extracts obtained from 253G1 *SALL3*$^{-/-}$ *DNMT3B1*$^{-/mut}$ cell.

Supplementary Figure 8. ChIP-seq analysis of SALL3 in 253G1 control shRNA cells.

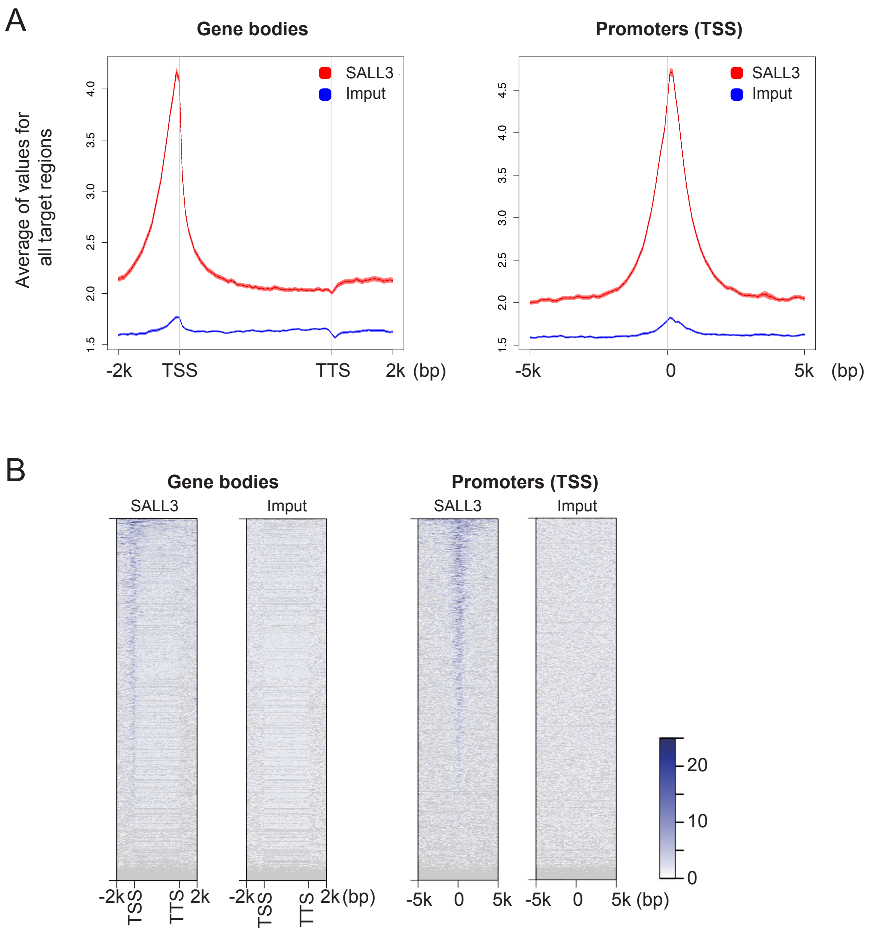

Supplementary Figure 8. ChIP-seq analysis of SALL3 in 253G1 control shRNA cells.
SALL3 binding distributions across gene bodies and promter regions in 253G1 control shRNA cells were determined as average plots (A) or as heatmap (B). Average plots and heatmap were generated with tag distributions (using bigWIG metrics) across gene bodies or promoter regions. TSS: transcription start sites, TTS: transcription termination sites.

Supplementary Figure 9. ChIP-seq analysis of DNMT3B in 253G1 *SALL3* KD cells.

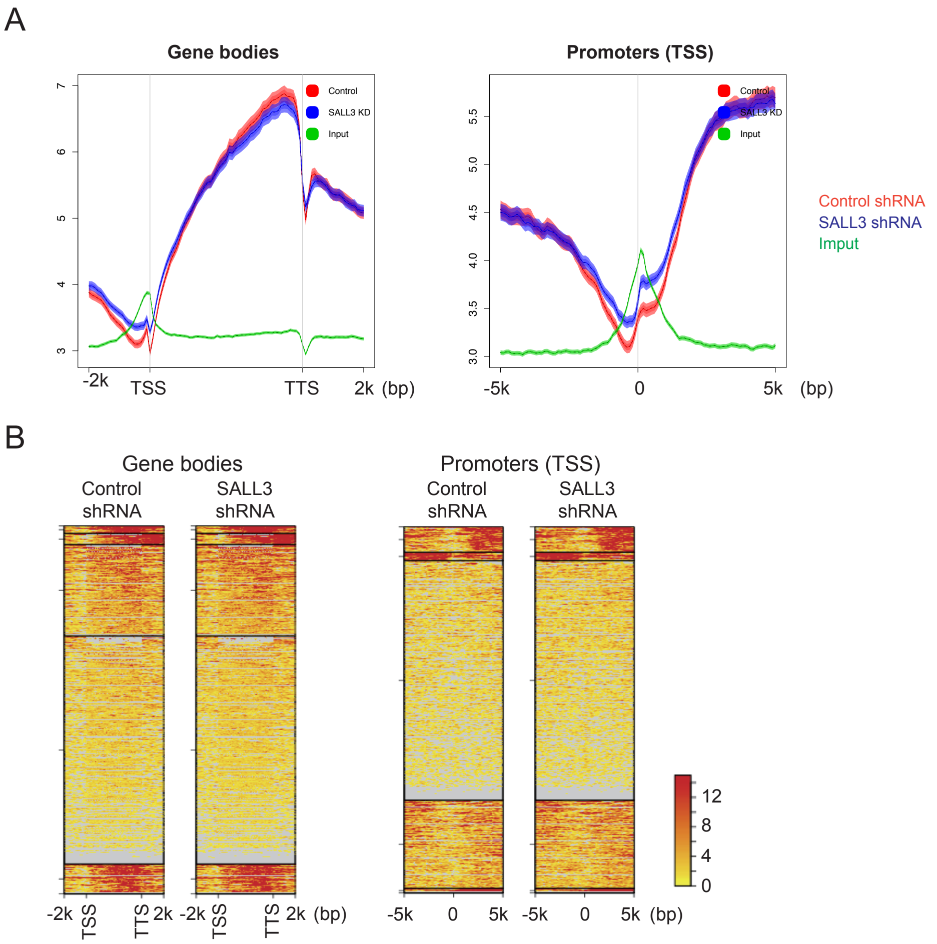

Supplementary Figure 9. ChIP-seq analysis of DNMT3B in 253G1 *SALL3* KD cells.
DNMT3B binding distributions across gene bodies and promter regions in 253G1 *SALL3* shRNA cells and 253G1 control shRNA cells were determined as average plots (A) or as heatmap (B). Average plots and heatmap were generated with tag distributions (using bigWIG metrics) across gene bodies or promter regions. TSS: transcription start sites, TTS: transcription termination site.

Supplementary Figure 10. Binding of DNMT3B to gene bodies is regulated by SALL3.

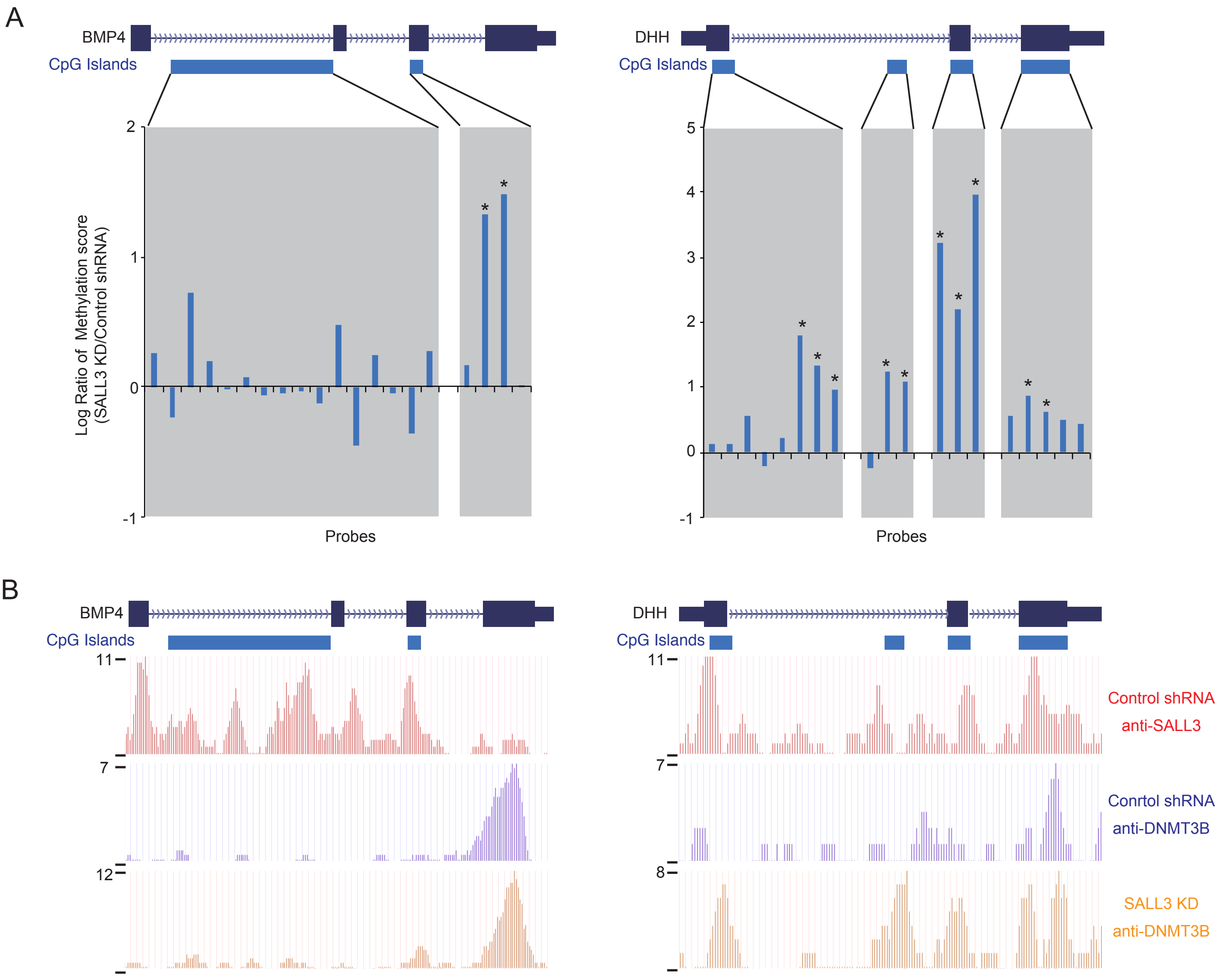

Supplementary Figure 10. Binding of DNMT3B to gene bodies is regulated by SALL3. (A)HumanMethylation450K BeadChip analysis of the BMP4 locus (left) and DHH locus (right). Top: schematic of a gene locus on the chromosome. Middle: schematic of a CpG island locus in two genes depicted by the UCSC Genome Browser. Bottom: methylation score. Gray vertical bars highlight CpG island regions (n = 3). Asterisks highlight probes with a significant difference in the methylation score between the control and SALL3 knockdown ($P < 0.05$, t-test). (B) The ChIP-seq data of the BMP4 locus (left) and DHH locus (right) are depicted by the UCSC Genome Browser. Top track: SALL3 protein binding to genomic regions in 253G1 control shRNA cells. Middle and bottom tracks: DNMT3B protein binding to genomic regions in 253G1 control shRNA cells and 253G1 SALL3 shRNA cells.

Supplementary Figure 11. Targeted deletion of *SALL3* in the hiPSC line 253G1.

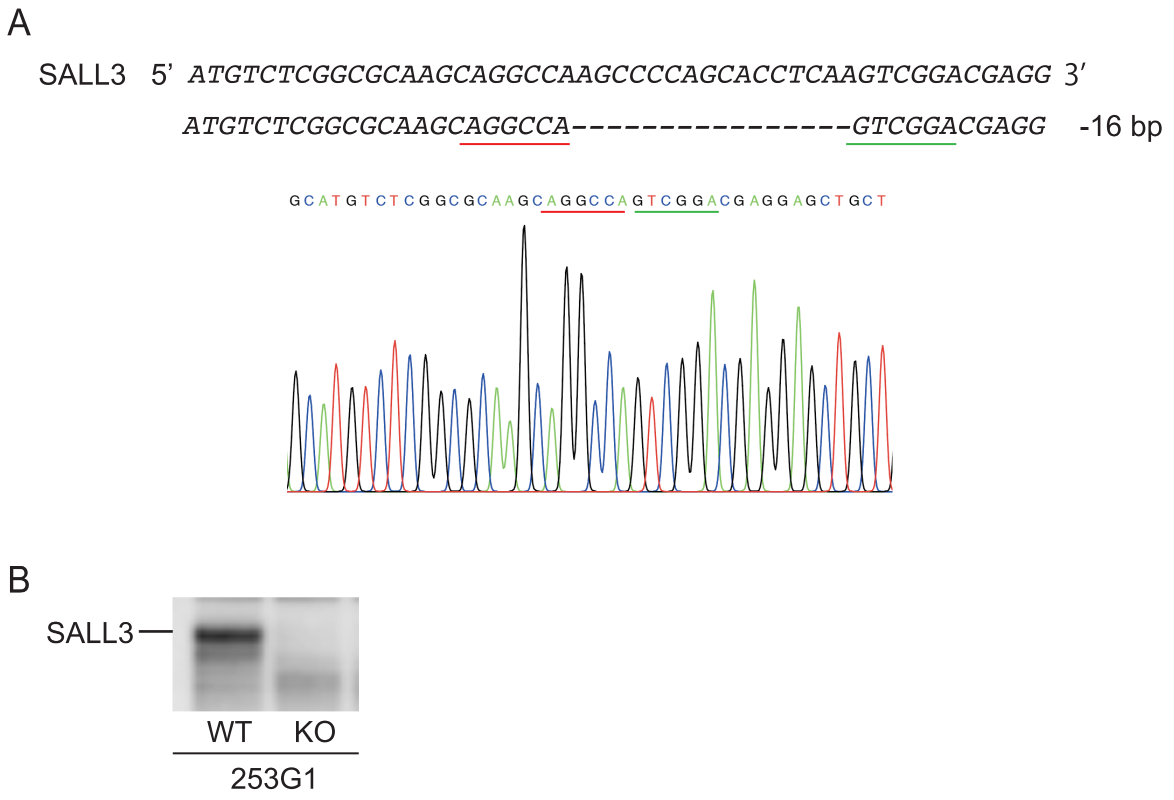

Supplementary Figure 11. Targeted deletion of *SALL3* in the hiPSC line 253G1.

(A) Sanger sequencing information for the targeted region of representative clone of SALL3 knockout 253G1 cells. The colored lines under the sequence correspond to the lines under sequencing peaks. (B) Western blot analysis of the total extracts obtained from *SALL3* knockout cells.

Supplementary Figure 12. Raw images of Western blots shown in main text.

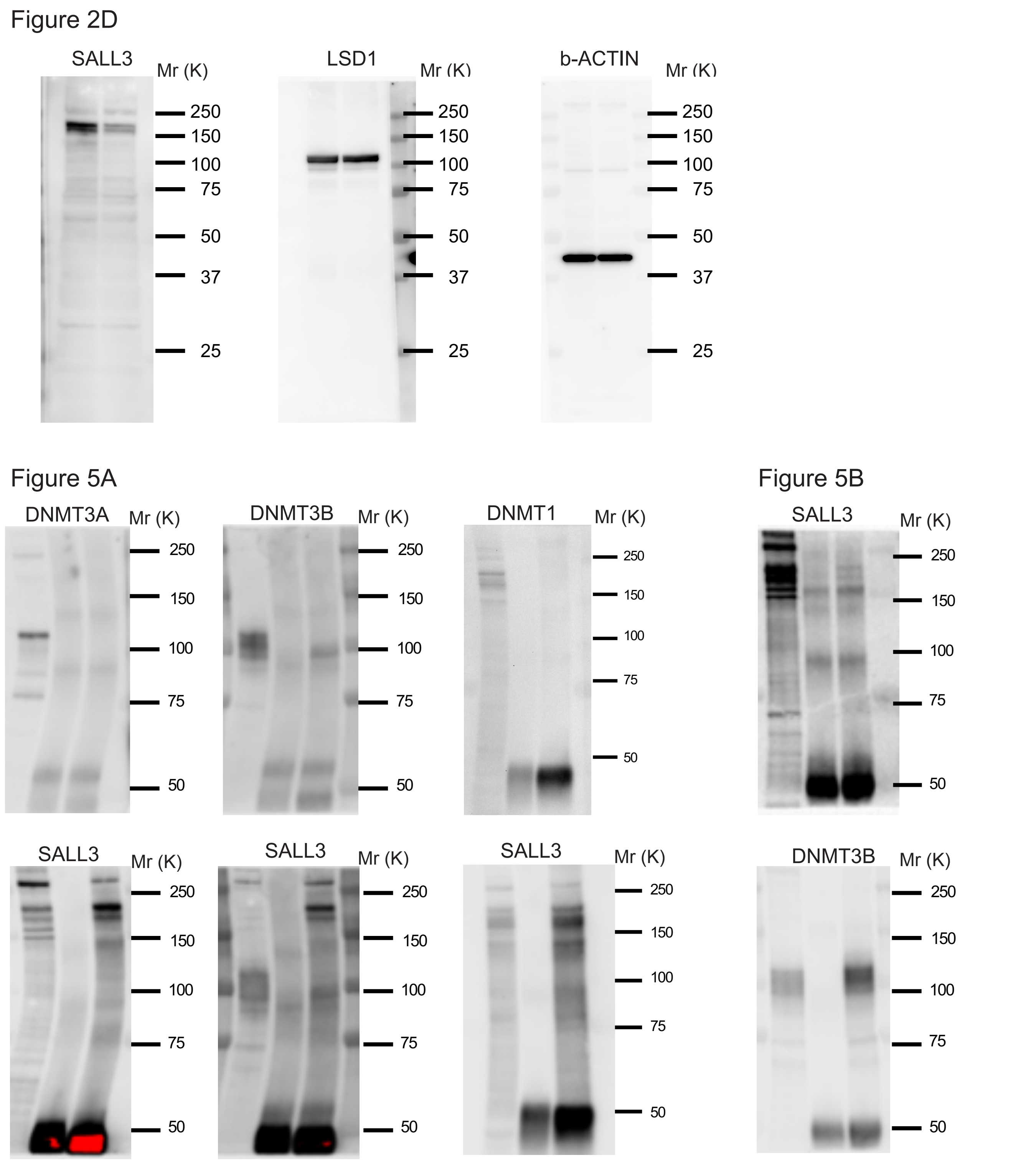

Supplementary Figure 12. Raw images of Western blots shown in main text.

Top panel shows the Western blots for Figure 2D and the bottom panels show the Western blots for Figure 5A and 5B. The molecular weight markers are indicated with Mr (K).

Supplementary Table 1. Bioinformatics pathway analysis of the genes containing hypermethylated probes in the gene body regions.

Pathway	-log(P-value)	Overlap	Molecules
Axonal Guidance Signaling	1.01E-10	18.2 % (83/457)	ABLIM1, ABLIM2, ADAM11, ADAM19, ADAMTS2, ADAMTS7, ADAMTS9, ARHGEF6, ARHGEF12, ARPC1B, BMP4, BMP7, BMP8A, BMP8B, C9orf3, CDK5, DPYSL5, ECE2, ECEL1, EFNA3, EPHA2, EPHA8, EPHA10, EPHB1, EPHB2, EPHB3, FZD5, GDF7, GLI2, GLI3, GNA14, GNAO1, HHIP, KLB, LIMK1, LINGO1, MYL5, NFATC4, NOTUM, NTF3, NTN1, NTNG2, NTRK1, NTRK2, NTRK3, PIK3R6, PITRM1, PLCB1, PLCB2, PLCD1, PLXND1, PRKAR1B, PRKCA, PRKCB, PRKCG, RHOD, ROBO3, SEMA3E, SEMA4A, SEMA6C, SHANK2, SLIT1, SRGAP3, SUFU, TUBA8, TUBA1A, TUBB3, TUBB4A, UNC5A, UNC5C, UNC5D, VEGFB, WNT1, WNT6, WNT11, WNT10A, WNT10B, WNT2B, WNT3A, WNT5A, WNT5B, WNT7B, WNT9B
Neuropathic Pain Signaling in Dorsal Horn Neurons	4.61E-07	24.3 % (28/115)	CAMK1D, CAMK2B, CAMK2D, GRIA3, GRIN1, GRIN2C, GRIN2D, GRIN3B, GRM1, GRM5, GRM6, ITPR1, KCNH2, KCNN4, KCNQ2, KCNQ3, KLB, NOTUM, NTRK2, PIK3R6, PLCB1, PLCB2, PLCD1, PRKAR1B, PRKCA, PRKCB, PRKCG, SRC
Basal Cell Carcinoma Signaling	5.13E-07	29.2 % (21/72)	APC2, BMP4, BMP7, BMP8A, BMP8B, FZD5, GLI2, GLI3, HHIP, SUFU, WNT1, WNT6, WNT11, WNT10A, WNT10B, WNT2B, WNT3A, WNT5A, WNT5B, WNT7B, WNT9B
Human Embryonic Stem Cell Pluripotency	5.12E-06	21.0 % (30/143)	BMP4, BMP7, BMP8A, BMP8B, FOXO1, FZD5, KLB, LEFTY1, NODAL, NTF3, NTRK1, NTRK2, NTRK3, PIK3R6, S1PR5, SMAD6, SPHK1, TGFB1, UTF1, WNT1, WNT6, WNT11, WNT10A, WNT10B, WNT2B, WNT3A, WNT5A, WNT5B, WNT7B, WNT9B
Molecular Mechanisms of Cancer	7.97E-06	15.5 % (61/394)	ADCY2, ADCY4, ADCY9, ARHGEF3, ARHGEF4, ARHGEF6, ARHGEF12, ARHGEF19, BMP4, BMP7, BMP8A, BMP8B, CAMK2B, CAMK2D, CDH1, CDK5, CDK6, CDK20, CDKN2C, CTNNA2, DHH, FOXO1, FZD5, GAB2, GNA14, GNAO1, HIPK2, IHH, KLB, LRP1, MAP2K6, MAPK13, PIK3R6, PLCB1, PLCB2, PRKAR1B, PRKCA, PRKCB, PRKCG, RALGDS, RASGRF1, RHOD, RHOF, RHOT2, SMAD6, SRC, SUFU, SYNGAP1, TAB1, TGFB1, WNT1, WNT6, WNT11, WNT10A, WNT10B, WNT2B, WNT3A, WNT5A, WNT5B, WNT7B, WNT9B

Suplementary Table 2. Informations about the hiPSC lines.

Cell Line	Trans gene	Method of reprograming	Cell sourse	Donor Age	Donor Sex	Passage# Microarray (in-house passage#)	Reference
201B7	OCT3/4, SOX2, KLF4, c-MYC	Retrovirus	Human dermal fibroblasts	36	Female	27	Takahashi K, et al. Cell. 2007 Nov 30;131(5):861-72.
253G1	OCT3/4, SOX2, KLF4	Retrovirus	Human dermal fibroblasts	36	Female	32	Nakagawa M, et al. Nat Biotechnol. 2008 Jan;26(1):101-6. Epub 2007 Nov 30.
409B2	OCT3/4, SOX2, KLF4, L-MYC, LIN28, p53-shRNA	Episomal vector	Human dermal fibroblasts	36	Female	42	Okita K, et al. Nat Methods. 2011 May;8(5):409-12.
mc-iPS	OCT3/4, SOX2, KLF4, c-MYC	Plasmid	Human adipose stem cells	40-65	Female	(14)	Jia F, et al. Nat Methods. 2010 Mar;7(3):197-9.
Tic	OCT3/4, SOX2, KLF4, c-MYC	Retrovirus	Human fetus lung cells (MRC-5)	–	Male	50	Fujioka T, et al. Hum Cell. 2010 Aug;23(3):113-8
ATCC-DYR0100hiPSc	OCT3/4, SOX2, KLF4, c-MYC	Retrovirus	Neonatal dermal fibroblasts	Newborn	Male	(5)	
ATCC-HYR0103hiPSc	OCT3/4, SOX2, KLF4, c-MYC	Retrovirus	Hepatic fibroblast	31	Male	(5)	
HiPS-RIKEN-1A	OCT3/4, SOX2, KLF4, c-MYC	Retrovirus	Umbilical cord-derived fibroblasts	–	Female	21	Fujioka T, et al. Hum Cell. 2010 Aug;23(3):113-8
HiPS-RIKEN-2A	OCT3/4, SOX2, KLF4, c-MYC	Retrovirus	Umbilical cord-derived fibroblasts	–	Male	21	Fujioka T, et al. Hum Cell. 2010 Aug;23(3):113-8
HiPS-RIKEN-12A	OCT3/4, SOX2, KLF4	Retrovirus	Umbilical cord-derived fibroblasts	–	Male	16	Fujioka T, et al. Hum Cell. 2010 Aug;23(3):113-8
606A1	OCT3/4, SOX2, KLF4, L-MYC, LIN28	Episomal vector	Cord blood	–	Female	–	
648A1	OCT3/4, SOX2, KLF4, L-MYC, LIN28, p53-shRNA	Episomal vector	Peripheral blood	30s	Male	–	

Supplementary Table 3. Probe and primer sequences.

Gene	FAM-TAMRA Probe sequences (5´ - 3´)	Forward primer sequences (5´- 3´)	Reverse primer sequences (5´- 3´)
AFP	CGCTACACCCTGAGCTTGGCACAGATC	CCTGCATTCTCTGATGACAAGTTC	GCTTCACAAGGTTAATGAGAAACTC
FOXA2	AGGGCTACTCCTCCGTGAGCAACATGA	GTCCGACTGGAGCAGCTACTA	GTACGTGTTCATGCCGTTCATC
GATA4	TGGACATAGCCCCACAGTTGACACACTC	CCGACACCCCAATCTCGATATG	CACAGATAGTGACCCGTCCCA
GATA6	CCACCTCTTCTAACTCAGATGATTGCAGCA	TGCTCTGGTAATAGCAATAATTCCA	TGAGGCTGTAGGTTGTGTTGTG
KDR	TACACTCACAGGCCGGCTCTTTCGCT	CCCAAATTCCATTATGACAACACAG	CTGGTTCTTCTAACGGGATATCTTC
LIN28	CGCATGGGGTTCGGCTTCCTGTCC	CACGGTGCGGGCATCTG	CCTTCCATGTGCAGCTTACTC
NCAM1	CACTGCCAGACGGGAGCCTGATCTCT	CCCCATCAGACACTATCTGGTC	TCAGCATTCCAGTCCAGGGA
NES	TCTGTAGGCCCTGTTTCTCCTGCTCCA	GGCAGCGTTGGAACAGAGG	CCTTCCAGGACCTGAGCGA
NKX2.5	CGCGGCTTCCTCCGCCGTCG	CCTAGAGCCGAAAAGAAAGAGC	CCGCTCCAGCTCATAGACC
PAX6	CCAGCCAGACCTCCTCATACTCCTGCAT	GGCAAATAACCTGCCTATGCAAC	ACTCCGCCCATTCACCGAA
SALL3	ACCTCAAGGTGCACATGGGGACACACA	CGGGCCTTCACCACTAAGG	GGGTTCTCCACAGACAGGC
SOX7	CTCCACTCCAACCTCCAAGCCCACCT	TCCCCGGCCACCTACCAC	GTCGAATTCATTGCGATCCATGTC
SOX17	CCTTCCACGACTTGCCCAGCATCTTG	CTGCACAACGCCGAGTTG	CTCTGCCTCCTCCACGAAG
T	TGAGCCACAATGCCAGCCCACCT	CCTTGGAATGCCTGCCCATC	AGGCTGGGGTACTGACTGG
TH	AGGACAAGCTCAGGAGCTATGCCTCACG	CGTGTCTGAGAGCTTCAGTGAC	ACGGGTCGAACTTCACGGA
TNNT2	CCAAAGCCCAGGTCGTTCATGCCCAA	GGCTGAAGATGGCCCAATGG	TCTCCATCGGGGATCTTGGG
