# Supplementary material for: SALL3 expression balance underlies lineage biases in human induced pluripotent stem cell differentiation
Source: Nat Commun. 2019 May 15;10:2175. doi: 10.1038/s41467-019-09511-4 (PMC6520385; doi:10.1038/s41467-019-09511-4)
Supplement: Supplementary file 3 — Reporting Summary [file 41467_2019_9511_MOESM3_ESM.pdf]

nature research

Corresponding author(s): Yoji Sato

Last updated by author(s): Feb 26, 2019

Reporting Summary

Nature Research wishes to improve the reproducibility of the work that we publish. This form provides structure for consistency and transparency in reporting. For further information on Nature Research policies, see Authors & Referees and the Editorial Policy Checklist.

Statistics

For all statistical analyses, confirm that the following items are present in the figure legend, table legend, main text, or Methods section.

n/a	Confirmed	
☐	☒	The exact sample size (*n*) for each experimental group/condition, given as a discrete number and unit of measurement
☐	☒	A statement on whether measurements were taken from distinct samples or whether the same sample was measured repeatedly
☐	☒	The statistical test(s) used AND whether they are one- or two-sided<br>*Only common tests should be described solely by name; describe more complex techniques in the Methods section.*
☒	☐	A description of all covariates tested
☐	☒	A description of any assumptions or corrections, such as tests of normality and adjustment for multiple comparisons
☐	☒	A full description of the statistical parameters including central tendency (e.g. means) or other basic estimates (e.g. regression coefficient) AND variation (e.g. standard deviation) or associated estimates of uncertainty (e.g. confidence intervals)
☐	☒	For null hypothesis testing, the test statistic (e.g. *F*, *t*, *r*) with confidence intervals, effect sizes, degrees of freedom and *P* value noted<br>*Give P values as exact values whenever suitable.*
☒	☐	For Bayesian analysis, information on the choice of priors and Markov chain Monte Carlo settings
☒	☐	For hierarchical and complex designs, identification of the appropriate level for tests and full reporting of outcomes
☒	☐	Estimates of effect sizes (e.g. Cohen's *d*, Pearson's *r*), indicating how they were calculated

Our web collection on statistics for biologists contains articles on many of the points above.

Software and code

Policy information about availability of computer code

Data collection	Real-time PCR system: Step one plus, StepOne software v2.3 (life technologies). Flow cytometry: S3 cell sorter, ProSort software (BioRad). Immunofluorescence staining analysis: BZ-X710 fluorescence microscope, BZ-X viewer software (Keyence). Western blot analysis: ChemiDoc Touch Imaging System, Image Lab software (BioRad).
Data analysis	Expression console software (Affymetrix), R statistics software (R 3.3.2), and SYSTAT 13 Software (Systat Software Inc.)

For manuscripts utilizing custom algorithms or software that are central to the research but not yet described in published literature, software must be made available to editors/reviewers. We strongly encourage code deposition in a community repository (e.g. GitHub). See the Nature Research guidelines for submitting code & software for further information.

Data

Policy information about availability of data

All manuscripts must include a data availability statement. This statement should provide the following information, where applicable:
- Accession codes, unique identifiers, or web links for publicly available datasets
- A list of figures that have associated raw data
- A description of any restrictions on data availability

All data available within this study are available from the corresponding author upon reasonable request. mRNA microarray and ChIP-seq data sets generated for this study are available from the NCBI GEO database under accession number GSE88963, GSE114977 and GSE104863.

Field-specific reporting

Please select the one below that is the best fit for your research. If you are not sure, read the appropriate sections before making your selection.

☒ Life sciences ☐ Behavioural & social sciences ☐ Ecological, evolutionary & environmental sciences

For a reference copy of the document with all sections, see nature.com/documents/nr-reporting-summary-flat.pdf

Life sciences study design

All studies must disclose on these points even when the disclosure is negative.

Sample size	Sample size was determined based on our previous experience and the work of other groups using human iPS cells as experimental materials.
Data exclusions	No data were excluded from the analysis.
Replication	All the experimental findings were reliably reproduced. The replication is indicated in corresponding Figure legends.
Randomization	This experimental design dose not contain a comparison between groups that needs to consider randomization.
Blinding	This experimental design dose not contain a comparison between groups that needs to consider blinding.

Reporting for specific materials, systems and methods

We require information from authors about some types of materials, experimental systems and methods used in many studies. Here, indicate whether each material, system or method listed is relevant to your study. If you are not sure if a list item applies to your research, read the appropriate section before selecting a response.

Materials & experimental systems

n/a	Involved in the study	
☐	☒	Antibodies
☐	☒	Eukaryotic cell lines
☒	☐	Palaeontology
☒	☐	Animals and other organisms
☒	☐	Human research participants
☒	☐	Clinical data

Methods

n/a	Involved in the study	
☐	☒	ChIP-seq
☐	☒	Flow cytometry
☒	☐	MRI-based neuroimaging

Antibodies

Antibodies used	anti-PAX6 (BioLegend PRB-278P-100, 1:200) , anti-Oct-3/4 (BD 611202, 1:200) antibody, anti-SALL3 (Abnova PAB28233, 1:100), anti-DNMT3B (R&D Systems AF7646, 1:100), anti-LSD1 (Cell Signaling Technology 2184, 1:1000), and anti-β-actin (Sigma-Aldrich A5441, 1:1000)
Validation	Immunofluorescence staining (human): anti-PAX6,

Eukaryotic cell lines

Policy information about cell lines

Cell line source(s)	HiPSC lines were obtained from RIKEN Cell Bank (201B7, 253G1, 409B2, 606A1, 648A1, HiPS-IKEN-1A, HiPS-RIKEN-2A, and HiPS-RIKEN-12A), the American Type Culture Collection (ATCC-DYR0110 hiPSC and ATCC-HYR01103 hiPSC), the JCRB Cell Bank (Tic), and System Biosciences (human mc-iPS).
Authentication	None of the cell lines used were authenticated.
Mycoplasma contamination	All cell lines were tested negative for mycoplasma contamination.
Commonly misidentified lines (See ICLAC register)	No commonly misidentified cell lines were used.

ChIP-seq

Data deposition

☒ Confirm that both raw and final processed data have been deposited in a public database such as GEO.

☒ Confirm that you have deposited or provided access to graph files (e.g. BED files) for the called peaks.

Data access links<br>*May remain private before publication.*	ChIP-seq data sets generated for this study are available from the NCBI GEO database under accession numberGSE104863.
Files in database submission	GSM2809093_1_01AS_005RTakara_Control_DNMT3B_hg19_i70_uniqnorm_signal.bw<br>GSM2809094_2_00YM_005RTakara_SALL3_KD_DNMT3B_hg19_i85_uniqnorm_signal.bw<br>GSM2809095_3_00D6_001YTakara_Input_hg19_i96_uniqnorm_signal.bw<br>GSM3258646_1_0395_00EUTakara_Control_SALL3_hg19_i90_uniqnorm_signal.bw<br>GSM3258647_2_03E7_001YTakara_Control_Input_hg19_i93_uniqnorm_signal.bw
Genome browser session<br>(e.g. UCSC)	Not applicable.

Methodology

Replicates	ChIP-seq: One replicate per sample.
Sequencing depth	SAMPLE, TOTAL READS, UNIQUE ALIGMENTS<br>Control_DNMT3B, 37670845, 32913487<br>SALL3_KD_DNMT3B, 40549229, 35760730<br>Input, 39488353, 33888634<br>Control_SALL3, 28657495, 23195235<br>Control_Input, 42560262, 35067264
Antibodies	Rabbit polyclonal anti-DNMT3B antibody (sc-20704, Santa Cruz Biotechnology) and anti-SALL3 antibody (PAB28233, Abnova) was used for ChIP.
Peak calling parameters	Genome build for alignments: hg19. Peak calling algorithm: SICER 1.1. SICER cutoff is FDR 1e-10 with gap parameter of 600bp. Peak filtering was performed by removing false ChIP-Seq peaks as defined within the ENCODE blacklist.
Data quality	SAMPLE, NUMBER OF PEAKS<br>Control_DNMT3B, 24307<br>SALL3_KD_DNMT3B, 21811<br>Control_SALL3, 3288
Software	Illumina Genome Analyzer II. Active Motif software.

Flow Cytometry

Plots

Confirm that:

☒ The axis labels state the marker and fluorochrome used (e.g. CD4-FITC).

☒ The axis scales are clearly visible. Include numbers along axes only for bottom left plot of group (a 'group' is an analysis of identical markers).

☒ All plots are contour plots with outliers or pseudocolor plots.

☒ A numerical value for number of cells or percentage (with statistics) is provided.

Methodology

Sample preparation	Cardiac differentiated hiPSCs were fixed using the BD Cytofix fixation buffer (BD Biosciences) for 20 min and permeabilized using BD Perm/Wash buffer (BD Biosciences) for 10 min at room temperature. The cells were incubated for 1 h at room temperature with mouse anti-cardiac troponin T monoclonal antibody (ab8295, Abcam). Indirect immunostaining was then completed with anti-mouse IgG Alexa Fluor 488-conjugated secondary antibody (A28175, Thermo Fisher Scientific) for 1 h. Normal mouse IgG antibody was used as a negative control (5415, Cell Signaling Technology). Stained cells were analyzed using an S3 cell sorter (BioRad). For flow cytometric analysis, live cells were gated using a SSC-area and FSC-area gate. Data retrieved from sorting was analyzed using FlowJo software (Tree Star).
Instrument	BIO-RAD, S3 cell sorter.
Software	FlowJo (Tree Star)
Cell population abundance	10,000 living cells were analyzed.

Gating strategy

The live cells were gated using a SSC-area and FSC-area gate.

☒ Tick this box to confirm that a figure exemplifying the gating strategy is provided in the Supplementary Information.
