# Supplementary material for: SALL3 expression balance underlies lineage biases in human induced pluripotent stem cell differentiation
Source: Nat Commun. 2019 May 15;10:2175. doi: 10.1038/s41467-019-09511-4 (PMC6520385; doi:10.1038/s41467-019-09511-4)
Supplement: Supplementary file 4 — Description of Additional Supplementary Files [file 41467_2019_9511_MOESM4_ESM.pdf]

Description of Additional Supplementary Files

File Name: Supplementary Data 1.

Description: A list of probes with significantly different expression levels among 10 hiPSC lines that defined filtering criteria (see Methods).

File Name: Supplementary Data 2.

Description: A list of lineage marker genes used for this study. Total 97 genes comprised of 45 ectoderm markers, 56 mesoderm markers and 27 endoderm markers, which included common markers for two or three germ layers.

File Name: Supplementary Data 3.

Description: Differentiation propensity marker candidate genes. There were 90 positive and 70 negative genes, 20 positive and 7 negative genes, and 7 positive and 25 negative genes for ectoderm, mesoderm, and endoderm differentiation markers, respectively.

File Name: Supplementary Data 4.

Description: A list of genes that have hypermethylated probes in the gene body region. We comprehensively compared the DNA methylation landscape of *SALL3* KD 253G1 cells with that of control shRNA-transduced 253G1 cells, using the Illumina Infinium HumanMethylation450 (HM450) DNA methylation array.

File Name: Supplementary Data 5.

Description: We compare the gene expression profile of SALL3 KD 253G1 cells with that of control shRNA-transduced 253G1 cells, using the mRNA array analysis. (Left side) A group of genes whose expression level was increased (log ratio >= 2) by SALL3 KD. (Right side) A group of genes whose expression level was decreased (log ratio <= 0.5) by SALL3 KD.
